# How to Help Microbes Save Us from PFAS

**DOI:** 10.1021/acscentsci.6c01102

**Published:** 2026-07-10

**Authors:** XiaoZhi Lim

## Abstract

To break down “forever chemicals”, bacteria and fungi need a way to avoid or evade their own toxic by-products.

## Graphical abstract

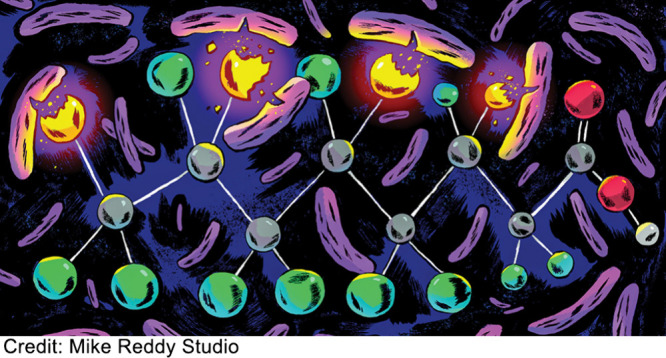

More than 1 million liters of aqueous film-forming foams (AFFFs) are stored across Ireland. At power plants, chemical refineries, airports, and pharmaceutical facilities, AFFFs lie in wait inside the pipes of fire-suppression systems.

**Figure:**
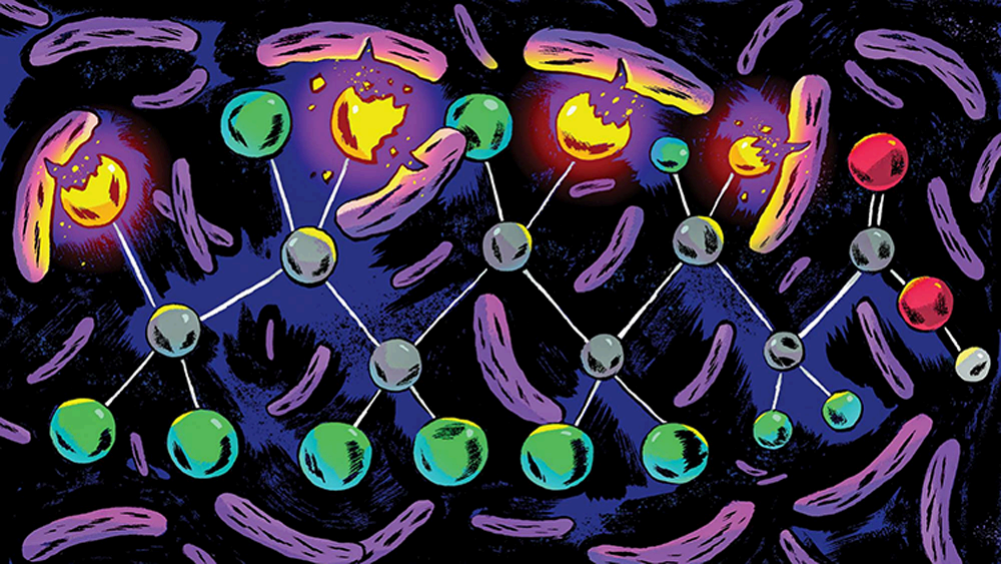
Bacteria, fungi, and scientists are getting creative in how they destroy fluorinated materials. Credit: Mike Reddy Studio.

But these foams contain some “forever chemicals”—also known as per- and polyfluoroalkyl substances (PFAS)—that are well-known toxins. Both the foams and their PFAS components may soon be banned in Europe. If the curtain falls on AFFFs, there are few good ways to get rid of them.

“There was no national mechanism to deal with this stored AFFF,” says Cormac Murphy, a microbiologist at University College Dublin. PFAS waste is currently destroyed by energy-intensive incineration at temperatures above 1,100 °C. In some places, such as Ireland, these high-temperature incinerators don’t exist, so any unwanted AFFFs will need a one-way ticket to mainland Europe, where they’ll meet their fiery end.

But Murphy hopes that a different fate could await them. In his laboratory, he’s growing a fungus, and last year, his team started feeding it with a chemically treated version of an AFFF.

It turns out that the carbon–fluorine bond, one of the strongest in organic chemistry, isn’t too strong for microbes to crack. Over the past 10 years or so, researchers working in settings from the wetlands of New Jersey to industrially contaminated soils in northern Portugal have turned up microbes that can defluorinate certain PFAS to some degree. None, however, can complete the job on their own.

Now researchers are realizing that, in addition to coaxing microbes to break the strong carbon–fluorine bond, they should also help the bugs avoid the fallout of their defluorination activity. Breaking down PFAS generates toxic products that can gum up microorganisms’ machinery or even kill them. Meanwhile, current PFAS degradation methods rarely yield energy or molecules that resemble microbial food, so researchers are also pursuing reactions that could offer some kind of benefit to microorganisms.

While researchers don’t yet have a clear microbial winner, they do have new strategies for helping microbes save us from PFAS. “There’re so many pieces that have to come together,” says Lawrence Wackett, a biochemist at the University of Minnesota.

## A tale of two labs

When Murphy moved his lab in 2015, physical chemist James Sullivan was just two floors down from him. Unbeknownst to each other, the two teams began working on the same problem: PFAS degradation. Sullivan’s team was using photocatalysis to break down the forever chemical perfluorooctanoic acid (PFOA), while Murphy’s team was feeding PFOA to a fungus, *Cunninghamella elegans*.

“I didn’t even know he was interested in this,” Sullivan says of Murphy’s work. Then Mohd Faheem Khan, who was at the time a postdoctoral researcher in Murphy’s lab, met Jhimli Paul Guin, who was then one of the postdocs in Sullivan’s lab, at a university event.

“They talked to each other, and they find out that they’re working on the same thing, except one’s doing chemistry, one’s doing biology,” says Murphy. “And then they came to us and said, ‘Could we do something together?’ ”

The collaboration would prove fortuitous for *Cunninghamella elegans*. In a previous study, the fungus mostly metabolized PFAS into a molecule—5:3 fluorotelomer carboxylic acid (FTCA)—that poisoned its enzymes and stopped the defluorination process early.

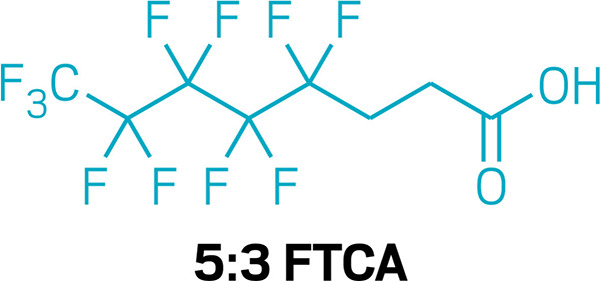



That is where Sullivan’s team came in with its photocatalytic treatment. His lab’s bismuth oxyiodide catalyst helped by chopping PFOA up into smaller pieces first, lowering the chances for 5:3 FTCA to form and allowing *Cunninghamella elegans* to carry out defluorination longer.

Paul Guin first treated PFOA with the photocatalytic process for 2 h and then handed off the intermediates to Khan, who fed them to *Cunninghamella elegans* for 2 days. The teams achieved 90% degradation and 60% defluorination with their two-stage approach, compared with just 35–40% degradation and 20–30% defluorination separately.

## The fluoride problem

Besides making 5:3 FTCA, defluorination produces a smaller yet more problematic poison: the fluoride ion itself. Most environmental researchers consider true detoxification of PFAS, whether by physical, chemical, or biological methods, to mean that the fluorine atoms have been separated from carbon. That would yield inorganic fluoride, which is commonly added to drinking water in the US and other countries.

Fluoride is not so innocuous for microbes, though. It binds to the metal centers in microbial enzymes, shutting down metabolism.

To Randy Stockbridge, a molecular biologist at the University of Michigan, surviving fluoride, rather than breaking the ultrastrong carbon–fluorine bond, could be the greater challenge for microbes. “We know that enzymes can do ridiculously difficult chemistry very fast,” Stockbridge says. “But I think it is a more complex situation if the fluoride that’s being produced by these reactions is so toxic [to microbes].”

To cope with naturally occurring low levels of fluoride in the environment, nearly every microbe has evolved some kind of mechanism to move fluoride ions outside their cells. Specifically, they have special proteins in their cell membranes called fluoride exporters.

Stockbridge reported one of these exporters in bacteria for the first time in 2012: an active ion pump dubbed CLC^F^. Later, she discovered another type of exporter in bacteria: a channel called Fluc that allows fluoride ions to flow in and out of cells and doesn’t require energy.

**Figure:**
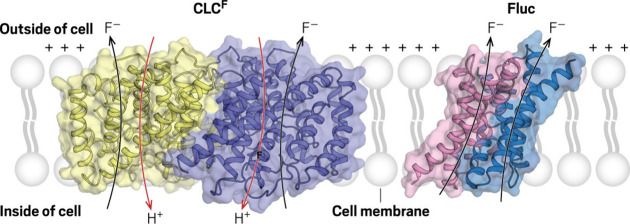
**Innate defense.** Because fluoride can be toxic in microbial cells, typical bacteria produce either the ion-channel protein CLC^F^, which actively pumps fluoride ions out of the cell, or Fluc, which allows fluoride ions to passively diffuse in either direction, depending on the concentration gradient. Credit: Randy Stockbridge.

Although CLC^F^ and Fluc worked okay in the preindustrial world, Minnesota’s Wackett became concerned that microbes may not have evolved to deal with the amount of fluoride that PFAS could release. If a bacterium tears up a single PFOA molecule, its cell gets jolted with up to 15 fluoride ions. On PFAS-rich diets, those ions can quickly reach lethal levels. “That’s going to be an enormous stress all inside the cell at once,” he says.

Wackett set out to test how important fluoride export is for PFAS-degrading bacteria. After Yujie Men from the University of California, Riverside, visited Wackett’s laboratory in 2022, the duo decided to do a proof of concept on Men’s microbes. They took an *Acetobacterium* culture that Men had showed could break down certain PFAS compounds, and they turned off the cells’ genes that code for Fluc.

The *Acetobacterium* lost its defluorinating ability, proving that the PFAS-degrading microbes need some way of getting rid of fluoride. “If there’s no fluoride ion exporter, there’s no defluorination activity,” Men says.

After that collaboration, Wackett’s group has been actively trying to engineer “an unnatural level of fluoride resistance” in *Pseudomonas* bacteria. One strategy is to double down on fluoride exporters by making a single organism express both CLC^F^ and Fluc exporters, which normally are not found in the same cell. He hopes that this approach could be a key for keeping microbes’ motors running while his team and others try to ramp up their other attributes, such as defluorination rate.

## Location, location, location

Fluoride would kill microorganisms if it were produced and stayed inside their cells. But what if defluorination reactions were to occur extracellularly?

Researchers such as Men are gradually realizing that indirect routes of defluorination could be more compatible with microorganisms. In other words, microbes could chew up a nonfluorinated part of a PFAS molecule while allowing the rest of that molecule to disintegrate outside the cell into fluoride ions and other pieces.

In 2023, Men’s group reported one of the highest degrees of defluorination of a group of PFAS by microbes—recovering some 80% of the fluorine content as fluoride ions. The researchers tested a mixture of microbes on chlorinated PFAS that are widely used in hydraulic fluids and lubricants. When they pieced the potential reaction pathways together, they found that the microbes hadn’t broken any carbon–fluorine bonds.

Instead, the microbes replaced the chlorine atoms with hydroxyl groups, which resulted in a highly unstable intermediate. Subsequently, the intermediate disintegrated and the fluoride ions fell off, seemingly without the microbe having to step in again.

**Figure:**
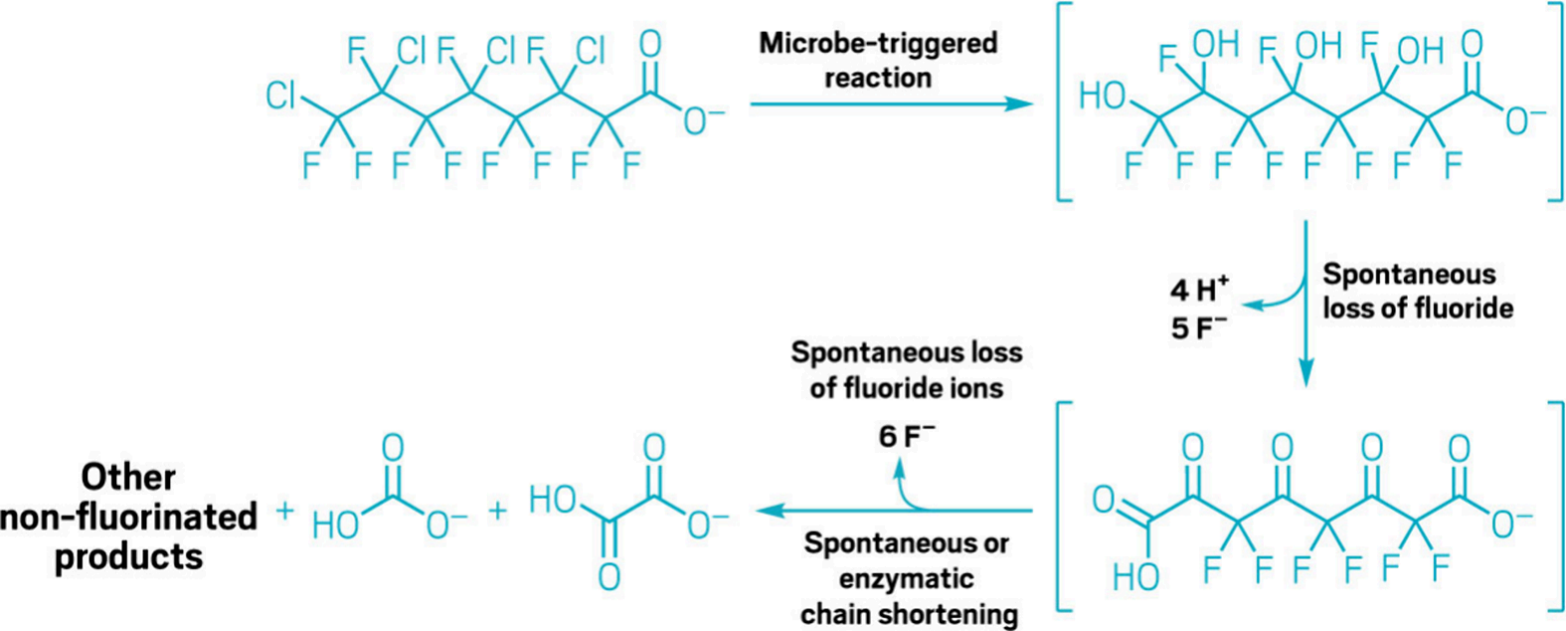
**Indirect defluorination.** Yujie Men’s group found that some microbes can break down chlorine-bearing molecules of per- and polyfluoroalkyl substances (PFAS) without breaking any C–F bonds. Instead, they replace the chlorine atoms with hydroxyl groups, which sets off a cascade of spontaneous reactions that ends in defluorination.

Men wonders whether the high degree of defluorination can be partly attributed to the fluoride ions being generated outside the cells. “If it’s outside, you don’t need to deal with the toxicity” as much, Men points out.

Wackett is now investigating whether microbes can pull off the blockbuster PFAS-destroying chemistry reported in 2022 by William Dichtel’s group at Northwestern University. That report got a lot of attention in part because it presented a low-temperature way for chemical reagents to break down PFOA and other perfluorinated carboxylic acids (PFCAs), some of the most concerning PFAS.

The key step is to boot a PFCA’s carboxylic acid group off as carbon dioxide. Once decarboxylated, the resulting PFAS intermediate was so unstable that it quickly jettisoned its fluorine atoms as fluorides. Wackett says microbes could replicate this step using enzymes called decarboxylases, which occur commonly in nature, to remove carboxylic acid groups.

Separately, Frank Loeffler’s lab group at the University of Tennessee is also trying to get microbes to perform the decarboxylation that Dichtel reported. Loeffler believes the reaction could reward the bugs for doing our dirty work. “If an organism figures this out, they actually get products like formate, acetate, oxalate,” Loeffler says. “Those are substrates for microbes to eat.”

## The next puzzles to solve

While the PFCAs are certainly a big prize for microbes to nab, Loeffler is also investigating fluorotelomer carboxylic acids, like the 5:3 FTCA that poisoned Murphy’s fungus. FTCAs may not be as notorious as PFOA, but researchers have found them in significant amounts. For example, 5:3 FTCA, which forms when discarded consumer products shed their stain-repellent coatings, is often the dominant PFAS in the fluids that leach out of landfills.

This year, Loeffler’s team observed that *Pseudomonas* bacteria could partially defluorinate some FTCAs but not others; they could even incorporate some FTCAs into their cell membranes. “I think these fluorotelomer carboxylic acids are important intermediates, and we need to understand what they do,” he says.

For practical remediation of PFAS, Men believes the bottleneck is processing speed: microbes simply cannot turn PFAS into fluoride fast enough. Her team is pursuing more-powerful enzymes that could degrade PFAS at a speed and scale meaningful for environmental remediation. But “it’s a long way to make it applicable,” she says.

Last summer, environmental microbiologist Serina Robinson and her team at the Swiss Federal Institute of Aquatic Science and Technology discovered an unusual source of enzymes that could break the carbon–fluorine bond: the human gut. Robinson had a hunch that, since humans consume fluorinated drugs, perhaps our gut microbes might have learned to break those down.

After searching a database of proteins found in gut microbes, Robinson’s team found more than 500 enzymes known to perform dehalogenation, all with similar ancestry. Several of these enzymes could break C–F bonds.

Fluorinated pharmaceuticals might have been an overlooked training ground for microbes that can transform PFAS. “The reason why we started working with [*Cunninghamella elegans*] initially was to look at fluorinated drugs,” says Murphy. The fungus was established as a good model of mammalian metabolism before Murphy started taking an interest in it.

For many years, Murphy’s lab used the fungus to help figure out what humans might metabolize drugs into, to avoid using animals in drug testing. It was the then postdoc Khan who suggested trying to find out whether *Cunninghamella elegans* could also work on something like PFAS, Murphy says.

But while Murphy’s and Sullivan’s teams achieved some success with their two-stage process to degrade PFOA, the actual AFFF material has been much more challenging to break down, Murphy says. A big reason is that AFFF is a complex mixture with many other molecules that the photocatalyst could act on, instead of targeting PFAS, Sullivan says.

The teams’ funding for their AFFF test ended in May, but they are working on new proposals to test their ideas for further improving the two-stage process—and to continue chipping away at the 20 L barrel of AFFF now sitting in Sullivan’s office that they bought for testing.

For now, Murphy and Sullivan are celebrating their collaboration, thanks to the serendipitous meeting of their former postdoc researchers. Paul Guin now works in industry, and Khan started his own research group. “You can’t design those kinds of interactions; they just happen,” Sullivan says.

*XiaoZhi Lim is a freelance contributor to* Chemical & Engineering News, *an independent news outlet of the American Chemical Society*.

